# Editorial: Pathophysiology of diabetic kidney disease

**DOI:** 10.3389/fcdhc.2026.1872162

**Published:** 2026-05-29

**Authors:** Oscar Lorenzo

**Affiliations:** Laboratory of Vascular Pathology and Diabetes, IIS Fundación Jiménez Díaz, Autónoma University, Spanish Biomedical Research Centre in Diabetes and Associated Metabolic Disorders (CIBERDEM) Network, Madrid, Spain

**Keywords:** biomarkers, diabetic kidney disease, inflammation, molecular mechanisms, precision medicine

Diabetic kidney disease (DKD) remains the leading global cause of chronic kidney disease, kidney failure, and premature mortality. DKD is not a single entity but a spectrum that includes non-albuminuric phenotypes, regressors, rapid progressors, and mixed forms. New population-level risk analyses and the recognition of rare complications have prompted a conceptual reclassification of the disease. At the same time, emerging mechanistic insights and the expanding capacity of biomarkers are reshaping diagnostic and prognostic frameworks, moving the field toward precision nephrology, where biological signatures guide individualized risk stratification and therapeutic decision−making.

The meta-analysis of DKD risk factors in Asia confirms the enduring relevance of classical determinants such as systolic blood pressure, HbA1c, diabetes duration, uric acid, creatinine, fasting glucose, and the waist-to-hip ratio. The strong association between diabetic retinopathy and DKD, even in biopsy-confirmed cases, reinforces the concept of systemic microangiopathy. Understanding microvascular dysfunction and associated cell-death may guide future investigations. Indeed, endothelial dysfunction, oxidative stress, RAAS activation, VEGF dysregulation, NLRP3 inflammasome activation, and altered renal hemodynamics represent critical mechanisms in DKD. Apoptosis, pyroptosis, ferroptosis, and autophagy complement this view, demonstrating how programmed cell death in podocytes, tubular epithelial cells, and mesangial cells marks a point of irreversible progression. In this context, the case of diabetic myonecrosis in end-stage kidney disease exemplifies the devastating complications of advanced diabetes and suggest that inflammation may play a central role in diabetic muscle ischemia. Interestingly, SGLT2 inhibitors, GLP-1 receptor agonists, and DPP-4 inhibitors may modulate these pathways, suggesting that future nephroprotection will increasingly target molecular mechanisms rather than clinical surrogates, an approach fully aligned with precision nephrology. Together, these findings reposition tubular injury, immune dysregulation, and programmed cell death as central drivers of DKD progression, shifting the focus away from the glomerulus alone and highlighting the need for mechanistic phenotyping.

Moreover, three studies provide complementary insights into the intersection of diabetes and kidney transplantation. Transplants using kidneys with pre-existing diabetic nephropathy may achieve acceptable long-term outcomes. Perioperative hyperglycemia and early insulin requirements appear modifiable and may improve the incidence and course of post-transplant diabetes. These observations underscore the potential for personalized peri−transplant metabolic management, another emerging application of precision nephrology.

In addition, one of the most compelling contributions of this collection is the identification of multiple biomarkers (metabolic, lipid-based, oxidative, immunologic) that refine risk prediction and disease mechanisms. The study of the non-HDL-C/HDL-C ratio (NHHR) reveals a nonlinear, L-shaped association with DKD risk, with a critical threshold at NHHR ≤ 2.82. Atherogenic dyslipidemia may therefore be not merely a cardiovascular risk factor but a direct modulator of renal vulnerability. Indeed, lipotoxicity injures podocytes and tubular cells, highlighting PCSK9-targeted therapies as promising nephroprotective strategies. Similarly, insulin-resistance markers (i.e., TyG, TyG-BMI, and TG/HDL-C) demonstrate strong predictive value for DKD, particularly in individuals with poor glycemic control. The sex-specific interaction observed for TG/HDL-C adds nuance and may guide more personalized risk assessment. In this line, lower bilirubin levels correlate with worse renal function, higher proteinuria, and poorer renal survival after DKD. Even subtle variations within the physiological range may carry prognostic significance. Along the same mechanistic axis, ACSL4, a ferroptosis-related enzyme, predicts rapid eGFR decline. AKT3 and FYN, linked to PANoptosis, show diagnostic value and correlate with GFR and BUN. MCUR1, CYP27B1, and G6PC, regulators of M2 macrophage polarization and efferocytosis, emerge also as key diagnostic genes for inflammation resolution. Finally, NONO, associated with MMP-9 and fibrosis, is strongly linked to poor renal prognosis. Therefore, these biomarkers illustrate how molecular phenotyping can identify high−risk subgroups, refine prognosis, and potentially guide targeted interventions-core components of precision nephrology.

Nevertheless, the biomarker thresholds, risk associations, and mechanistic findings may not be directly generalizable across different ethnicities, healthcare settings, or stages of CKD. These limitations underscore the need for validation in diverse cohorts before these markers can be fully integrated into precision−based clinical practice.

In conclusion, DKD is no longer an inevitable late complication of diabetes. It is transitioning from a clinically defined condition based on albuminuria and eGFR to a biologically stratified disease characterized by molecular signatures, immune pathways, and microvascular dysfunction. A new generation of biomarkers may enable earlier detection and more personalized intervention, marking a decisive step toward precision approaches to DKD management.

